# Association of Chronic Hyperglycemia With the Risk of Urolithiasis

**DOI:** 10.7759/cureus.47385

**Published:** 2023-10-20

**Authors:** Nidhal R Almuhanna, Abdullah M Alhussain, Reem B Aldamanhori, Qusay A Alabdullah

**Affiliations:** 1 Department of Urology, Imam Abdulrahman Bin Faisal University, Dammam, SAU

**Keywords:** hyperglycemia, glycemic control, insulin resistance, urolithiasis, diabetes mellitus

## Abstract

Background

The incidence of urolithiasis is increasing along with elevated rates of chronic hyperglycemia. Therefore, this study aimed to assess the association between high hemoglobin Alc (HbAlc) levels, in the form of type 2 diabetes mellitus (T2DM), and the risk of kidney stone formation among those living in the Eastern Province of Saudi Arabia.

Methodology

We conducted a cross-sectional study on a total of 501 patients with known cases of urolithiasis who visited King Fahad University Hospital (Khabar, Saudi Arabia). We calculated odds ratios (ORs) of having stones with respect to three parameters, namely, fasting blood glucose level, random blood glucose level, and glycosylated HbA1c testing.

Results

Of the 501 cases with urinary stones included in this study, the majority (223, 44.5%) were 41-59 years of age, and 350 (69.9%) were males. Our results showed that T2DM was significantly associated with high stone burden, with increased fasting plasma glucose, increased random blood glucose, and increased HbA1c being strong predictors. The significant associations between glycemic control measures and the risk of urolithiasis remained even after adjusting for factors related to insulin resistance.

Conclusions

According to our results, glycemic control can be an independent risk factor for urolithiasis. This critical finding demonstrates the need for further studies to investigate this particular group of patients.

## Introduction

Although stones in urolithiasis can be as small as the tip of a pin and nearly invisible to the naked eye, these tiny crystals can cause serious pain. The recrudescence of kidney stones as part of urolithiasis is estimated to be 3%-4% in women and 6%-9% in men globally [1]. Although stone formation has been associated with multifactorial causes, a link between increased stone formation and blood hyperglycemic states related to diabetes has been reported [2]. In addition to pain, hospitalizations, interventions, and missed workdays related to urolithiasis result in significant financial costs [3]. Urolithiasis is reportedly becoming more common in different parts of the world [4], concurrent with an increasing prevalence of type 2 diabetes mellitus (T2DM), obesity, and metabolic syndrome [5]. Based on recent studies and systematic reviews, increased prevalence and incidence of urolithiasis are evident across all age, race, and gender groups, despite the fact that these are considered non-modifiable risk factors [6,7].

Generally, the most common factors that influence stone formation are either genetic or environmental, with the influence of the former occurring at a slower rate compared to the latter [8]. Therefore, genetic factors are unlikely to be the cause of stone formation, as the influence of environmental changes occurs more rapidly. Stone formation is even more difficult to understand because the associated environmental factors have numerous and intricate risk variables, which add to the complexity of the situation. However, their impact is more noticeable than that of genetic factors because changes in them occur more frequently and their effects are often felt sooner. For example, a significant link between food and lifestyle choices and urolithiasis has been reported in epidemiological studies [9,10]. Increased weight, dietary factors, medications, family history, and some medical conditions (e.g., primary hyperthyroidism, hypertension, obesity, and diabetes) are considered major risk factors for stone formation and prevalence [11-13]. In addition, advancements and increased use of diagnostic imaging technology are other potential factors for this increase [14]. Regardless, the myriad causes and factors of stone formation demand further investigation, particularly its association with chronic hyperglycemia. Studies have provided pathophysiologic explanations and statistics showing the exact process and causes of stone formation, with several reporting influencing factors.

Recent studies attempting to provide pathophysiological explanations for the increased incidence of urolithiasis in diabetes worldwide suggest that insulin resistance has received the most attention as a potential risk factor [15,16]. The development of uric acid and calcium stones is facilitated by insulin resistance, as the latter is linked to abnormalities in renal ammonium production, increased urine acidity, hypocitraturia, and hypercalciuria [17-19], all of which potentially lead to stone formation [20,21].

In this retrospective cross-sectional study, we aimed to assess the association between high blood glucose levels (in the form of diabetes mellitus) and the risk of stone formation among those living in the Eastern Province of Saudi Arabia. Although there is strong epidemiological evidence linking diabetes to urolithiasis, not much is known about how the severity of diabetes and glycemic control may affect the likelihood of developing kidney stones. Therefore, we hope to highlight and emphasize the correlation between hyperglycemia in T2DM, as well as its severity, and the development of urolithiases in our study population.

## Materials and methods

Study duration, setting, and sampling

We performed this retrospective cross-sectional study at King Fahad University Hospital (KFUH), Khobar, Saudi Arabia. The study included 501 participants who were diagnosed with urolithiasis from January 2015 to December 2022. We obtained the data from the electronic hospital records of KFUH using QuadraMed, with patients chosen based on our inclusion and exclusion criteria. All patient information remained confidential throughout the entire study.

Study subjects and design

This retrospective cross-sectional study was conducted to evaluate the association between chronic hyperglycemia and risk of urolithiasis in KFHU patients meeting the following inclusion criteria: previous urolithiasis diagnosis, current urolithiasis diagnosis, T2DM patients of both genders with or without complications, and ≥30 years of age. We excluded patients <30 years of age inside or outside KFHU, as well as those with T1DM.

Study tool

We collected the data using a multivariable data collection sheet that included the following parameters to assess the severity of diabetes: insulin use, oral hypoglycemic use, and all diabetic complications, including retinopathy, diabetic foot, stroke, nephropathy, neurogenic bladder, neuropathy, heart attack, and peripheral artery disease. Glycemic control was assessed according to mainly glycated hemoglobin, as well as fasting blood glucose (FBG) and random blood glucose (RBG). Patients were screened based on their urolithiasis diagnosis and then assessed for their blood glucose and hemoglobin AlC (HbA1c) levels. The patients were then categorized into the following three groups: the major urolithiasis group, in which stones formed without evidential cause and blood glucose levels were within the normal range (HbA1c < 5.7%); the major urolithiasis group, in which stones formed in known cases of controlled T2DM (5.7% < HbA1c < 6.5%); and the major urolithiasis group, in which stone formed in known cases of uncontrolled T2DM (HbA1c > 6.5%) Finally, every stone was classified and organized according to the site and quantity (single/multiple), as well as high burden (>5 mm stone or multiple stones) or low burden (<5 mm single stone).

Data analysis

Data analysis was performed using SPSS version 26 (IBM Corp., Armonk, NY, USA). All categorical variables are presented as frequencies and percentages. Furthermore, we used the chi-square test to determine associations between variables. We performed univariate analysis to determine the odds ratios (ORs) and 95% confidence intervals (CIs) of high stone burden among different groups. Moreover, we used multivariate analysis to calculate the ORs of high stone burden after adjusting for age and sex, with statistical significance set at p-values <0.05.

## Results

A total of 501 cases with urinary stones were included in this study. The majority (223, 44.5%) of cases were 41-59 years of age, followed by 30-40 years of age (168, 33.5%). The gender distribution showed a male predominance; out of the 501 total cases, 350 (69.9%) were male patients (Table 1).

**Table 1:** Age and sex distribution (n = 501).

		Frequency (n)	Percentage (%)
Age (years)	30–40	168	33.5
	41–59	223	44.5
	>60	110	22
Sex	Male	350	69.9
	Female	151	30.1

Table 2 presents the patient information regarding diabetes. Most cases were non-diabetic (343, 68.5%) whereas 158 (31.5%) had T2DM. Overall, 100 (63.3%) cases were using oral hypoglycemics, 10 (6.3%) were using insulin, and 48 (30.4%) were using both types of medications. Diabetic complications were found in 41 (25.9%) cases. Out of 158 diabetic cases, 105 (66.5%) presented with uncontrolled T2DM. FBG levels of <100 mg/dL, 100-125 mg/dL, and >125 mg/dL were found in 348 (69.5%), 86 (17.2%), and 67 (13.4%) cases, respectively. RBG levels of <140 mg/dL, 140-199 mg/dL, and >199 mg/dL were found in 348 (69.5%), 103 (20.6%), and 50 (10%) cases, respectively. An HbA1c level of <5.7% was found in 316 (63.1%) cases.

**Table 2:** Diabetes mellitus parameters (n = 501). T2DM = type 2 diabetes mellitus; HbA1c = hemoglobin A1c

		Frequency (n)	Percentage (%)
Diabetes	T2DM	158	31.5
	Non-diabetic	343	68.5
Hypoglycemic medications use	Oral hypoglycemic	100	63.3
	Insulin use	10	6.3
	Both	48	30.4
Diabetes complications	Yes	41	25.9
	No	117	74.1
Fasting plasma glucose (mg/dL)	<100	348	69.5
	100–125	86	17.2
	>125	67	13.4
Random blood glucose (mg/dL)	<140	348	69.5
	140–199	103	20.6
	>199	50	10
HbA1c level	<5.7%	316	63.1
	5.7–6.4%	76	15.2
	>6.4%	109	21.8
T2DM control status	Uncontrolled T2DM	105	66.5
	Controlled T2DM	53	33.5

Table 3 presents the characteristics of participants’ urinary stones. Out of 501 cases, 246 (49.1%) presented with a single urinary stone, and 255 (50.9%) presented with multiple stones. Among these cases, 360 (71.9%) participants reported renal stones, 98 (19.6%) reported an upper ureteral stone, 42 (8.4%) reported a middle ureteral stone, 74 (14.8%) reported a lower ureteral stone, 58 (11.6%) reported a vesicoureteral junction stone, and nine (1.8%) reported a bladder stone.

**Table 3:** Characteristics of urinary stones (n = 501).

		Frequency (n)	Percentage (%)
Number of stones	Single	246	49.1
	Multiple	255	50.9
Location of the urinary stone	Renal stone	360	71.9
	Upper ureteral stone	98	19.6
	Middle ureteral stone	42	8.4
	Lower ureteral stone	74	14.8
	Bladder stone	9	1.8
	Vesicoureteral junction stone	58	11.6

**Figure 1:**
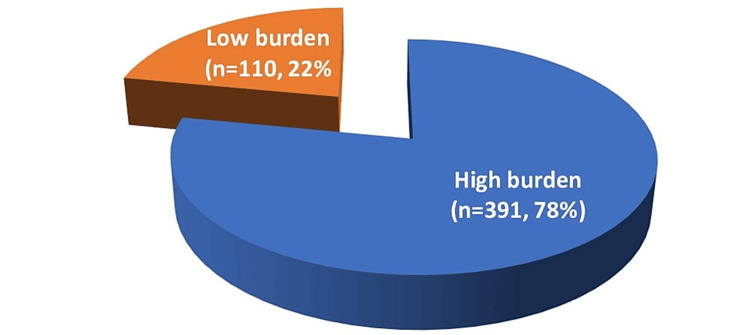
Burden of urinary stones (n = 501).

Table 4 presents the distribution of urinary stones. Most cases reported single stones in all locations. Out of 501 cases, 391 (78%) were found to have a high stone burden, and 110 (22%) cases were found to have a small stone burden, as seen in Figure 1.

**Table 4:** Distribution of urinary stones (n = 501).

Location	Number of stones	
	Single	Multiple
Kidney stone	187 (51.9%)	173 (48.1%)
Upper ureteral stone	90 (91.8%)	8 (8.2%)
Middle ureteral stone	40 (95.2%)	2 (4.8%)
Lower ureteral stone	73 (98.6%)	1 (1.4%)
Bladder stone	9 (100%)	
Vesicoureteral junction stone	56 (96.6%)	2 (3.4%)

Table 5 presents the results of the chi-square test between patient factors and urinary stone burden. T2DM was significantly associated with a high stone burden (136, 86.1%) compared with non-diabetic patients (255, 74.3%; p = 0.003). Increased (>125 mg/dL) fasting plasma glucose was significantly associated with a high stone burden (79, 91.9%; p = 0.001), as was increased (>199 mg/dL) RBG (43, 86%; p = 0.25) and increased (>6.4%) HbA1c (94, 86.2%; p = 0.009).

**Table 5:** Association between patient factors and urinary stone burden (n = 501). T2DM = type 2 diabetes mellitus; HbA1c = hemoglobin A1c

		Stone burden		P-value
		High	Low	
Age (years)	30–40	125 (74.4%)	43 (25.6%)	0.133
	41–59	173 (77.6%)	50 (22.4%)	
	>60	93 (84.5%)	17 (15.5%)	
Sex	Male	268 (76.6%)	82 (23.4%)	0.225
	Female	123 (81.5%)	28 (18.5%)	
Diabetes	T2DM	136 (86.1%)	22 (13.9%)	0.003
	Non-diabetic	255 (74.3%)	88 (25.7%)	
Diabetes complications	Yes	35 (85.4%)	6 (14.6%)	0.9
	No	101 (86.3%)	16 (13.7%)	
Fasting plasma glucose (mg/dL)	<100	257 (73.9%)	91 (26.1%)	0.001
	100–125	79 (91.9%)	7 (8.1%)	
	>125	55 (82.1%)	12 (17.9%)	
Random blood glucose (mg/dL)	<140	260 (74.7%)	88 (25.3%)	0.025
	140–199	88 (85.4%)	15 (14.6%)	
	>199	43 (86%)	7 (14%)	
HbA1c level	<5.7%	233 (73.7%)	83 (26.3%)	0.009
	5.7%–6.4%	64 (84.2%)	12 (15.8%)	
	>6.4%	94 (86.2%)	15 (13.8%)	
T2DM control status	Uncontrolled T2DM	91 (86.7%)	14 (13.3%)	0.965
	Controlled T2DM	46 (86.8%)	7 (13.2%)	

The univariate analysis revealed that the following factors were significant predictors of high stone burden: patient age >60 years (OR = 1.88; 95% CI = 1.01-3.51; p = 0.046), T2DM (OR = 2.13; 95% CI = 1.28-3.56; p = 0.004), fasting plasma glucose of 100-125 mg/dL (OR = 4; 95% CI = 1.78-8.97; p = 0.001), RBG >199 mg/dL (OR = 2.08; 95% CI = 1.09-3.61; p = 0.025), and HbA1c level >6.4% (OR = 2.23; 95% CI = 1.23-4.07; p = 0.009), as seen in Table 6.

**Table 6:** Univariate analysis of patient factors and odds of having a high stone burden (n = 501). T2DM = type 2 diabetes mellitus; HbA1c = hemoglobin A1c

		OR (95% Cl)	P-value
Age (years)	30–40	1.00 (referent)	-
	41–59	1.19 (0.75–1.9)	0.466
	>60	1.88 (1.01–3.51)	0.046
Sex	Male	1.00 (referent)	-
	Female	1.34 (0.83–2.17)	0.226
Diabetes	Non-diabetic	1.00 (referent)	-
	T2DM	2.13 (1.28–3.56)	0.004
Fasting plasma glucose (mg/dL)	<100	1.00 (referent)	-
	100–125	4 (1.78–8.97)	0.001
	>125	1.62 (0.83–3.17)	0.156
Random blood glucose (mg/dL)	<140	1.00 (referent)	-
	140–199	1.99 (0.9–4.79)	0.086
	>199	2.08 (1.09–3.61)	0.025
HbA1c level	<5.7%	1.00 (referent)	-
	5.7%–6.4%	1.9 (0.98–3.7)	0.059
	>6.4%	2.23 (1.23–4.07)	0.009

Another multivariate analysis was done after adjusting for age and sex. T2DM, a fasting plasma glucose level of 100-125 mg/dL, RBG >199 mg/dL, and an HbA1c level of >6.4% remained significantly associated with high urinary stone burden, as seen in Table 7.

**Table 7:** Multivariate analysis of patient factors and odds of having a high stone burden (n = 501). T2DM = type 2 diabetes mellitus; HbA1c = hemoglobin A1c

		OR (95% Cl)	P-value
Diabetes	Non-diabetic	1.00 (referent)	-
	T2DM	1.94 (1.1–3.56)	0.03
Fasting plasma glucose (mg/dL)	<100	1.00 (referent)	-
	100–125	3.74 (1.6–8.8)	0.003
	>125	1.55 (0.8–3.2)	0.223
Random blood glucose (mg/dL)	<140	1.00 (referent)	-
	140–199	1.8 (0.9–3.3)	0.07
	>199	1.85 (0.8–4.4)	0.16
HbA1c level	<5.7%	1.00 (referent)	-
	5.7%–6.4%	1.8 (0.9–3.6)	0.094
	>6.4%	2.1 (1.1–4.01)	0.035

## Discussion

We found associations between increased HbA1c, RBG, and FBG and the risk of urinary stone formation. Moreover, we found strong associations between urolithiasis and the glycemic control measures FBG and HbA1c, which remained even after readjusting for diverse confounders.

Based on our results, the highest odds of having urinary stones were seen in those with poor glycemic control and high insulin resistance. Our results also showed notable variations in urolithiasis incidence between males and females, with most subjects being male (69.9%). Approximately one-third (31.5%) of the patients diagnosed with urolithiasis were known to have diabetes, with similar results reported in multiple similar studies in different countries. Our analysis highlighted the importance of glycemic control to decrease the risk of urolithiasis, as our results suggested that the development of urolithiasis was more common in diabetic patients with poor glycemic control compared with the control group and other diabetic cases.

Unexpectedly, our results showed that the presence of diabetic complications was not a predictor of urolithiasis, as few cases had diabetic complications, as seen in some studies [22]. In contrast, a study done at Stanford University on the association between T2DM and other metabolic syndromes and urolithiasis concluded that the risk of urolithiasis was higher in T2DM patients with more severe forms of the disease [14]. In another population-based study from the United States, a random sample of urolithiasis patients was compared based on a prior diagnosis of T2DM, hypertension, and obesity. The study concluded that T2DM might be a risk factor for the development of uric acid stones. Moreover, the same association has been linked to hypertension in multiple studies [23,24]. Previous studies have shown that excretion of urinary calcium was positively associated with urinary glucose excretion in diabetic subjects. It is thought that glucose in urine due to poor glycemic control elevates calcium levels in urine and eventually causes calcium stone formation [22-26].

Regarding the strengths of this study, we took certain actions to increase the accuracy of the results. For example, stone diagnosis was supported by computed tomography scanning and ultrasonography. Moreover, the availability of glycemic control markers such as FPG and HbA1c allowed us to explore the relationship between hyperglycemia and the risk of kidney stones, which is challenging with a traditional questionnaire-based assessment.

Application

Several pernicious health issues are related to poor glycemic control [27]. Our study highlighted the association between uncontrolled blood sugar and urolithiasis. Our results suggest that the incidence of urolithiasis in diabetic patients can be decreased by improving their blood glucose control. We did not consider many factors that may increase the incidence of urolithiasis in this study. Even though the data were taken via blind sampling to ensure that confounders minimally affected the results, repeating the study could potentially lead to slight variation.

Limitations and recommendations

This study had some limitations. There may have been bias in the data, as they were gathered from a small midwestern community that is predominately Saudi. The local population’s socio-demographic data are similar to those of the Saudi eastern population overall. Additionally, the body mass index of the patients, which is an important risk factor for urolithiasis, could not be calculated due to the limited number of contactable patients [28,29]. Moreover, dietary information, which is also a strong risk factor for urolithiasis, could not be assessed [30]. Our healthcare system faces significant direct and indirect costs related to urolithiasis. Primary care physicians, nephrologists, and urologists could help to reduce the prevalence of the condition if they are able to recognize and treat underlying risk factors for forming stones. The results of this study suggest that further preventive measures are required for diabetic patients. Moreover, other possible risk factors should be studied to limit the suffering of the patients, as there is a growing incidence of T2DM locally.

## Conclusions

The current study demonstrated that hyperglycemia was linked to an increased incidence of kidney stones. We offer helpful information on the risk assessment of kidney stones from a pragmatic standpoint. Furthermore, glycemic management appeared to have a separate impact on the risk of kidney stones. This discovery may help to clarify the complicated etiopathogenesis of kidney stones in relation to diabetes. In fact, T2DM severity was found to be a significant risk factor for kidney stone disease, as determined by glycemic control. To verify our conclusions, future studies comparing T2DM treatment approaches should try to include kidney stones as an outcome of interest.
